# Editorial: Investigating VR in sports training: cognitive and performance impacts

**DOI:** 10.3389/fspor.2026.1937195

**Published:** 2026-08-03

**Authors:** Florian Heilmann, Robert Stojan, Dan Bürger, Sebastian Merker, Kerstin Witte

**Affiliations:** 1 Department of Movement Science, Institute of Sport Science, Martin-Luther University Halle-Wittenberg, Halle (Saale), Germany; 2 Faculty for Humanities, Institute III: Sport Science, Department of Sports Engineering/Movement Science, Otto von Guericke University Magdeburg, Magdeburg, Germany; 3 Institute for Applied Training Science, Leipzig, Germany

**Keywords:** athlete development, coaching, motor control, perceptual-cognitive skills, performance enhancement, sports science, virtual reality

## 1 Introduction

How can perceptual, cognitive, and motor skills be accurately assessed and effectively trained when real-world testing and practice are constrained by time, safety, logistical challenges, or environmental variability? Virtual reality (VR) has emerged as a promising answer to this challenge. Advances in immersive technologies, motion tracking, and real-time interaction now allow researchers and practitioners to simulate sport-specific demands with increasing ecological validity while maintaining the experimental control required for rigorous scientific investigation. VR has evolved from experimental laboratory technology into an increasingly valuable complement to traditional sports training.

The studies assembled in this Research Topic illustrate an important transition in the field. Rather than asking whether VR can be applied in sports, they examine when, how, and under which conditions immersive technologies can meaningfully enhance sport-specific perceptual–cognitive performance, motor control, and psychological skills. Together, these interdisciplinary contributions reveal both the considerable potential of VR and the methodological, technological, and practical challenges that must be addressed before its widespread implementation in everyday training environments. Importantly, they also provide valuable insights for coaches and practitioners by identifying situations in which VR can complement conventional training, create representative practice environments, and support more individualized approaches to athlete development.

## 2 VR training to improve motor control, balance, and sensorimotor adaptation

Balance and postural control are fundamental aspects of athletic performance, providing the foundation for efficient sensorimotor coordination during complex actions such as jumping, landing, sprinting, and changes of direction, while simultaneously reducing the risk of sports-related injuries. The contributions in this Research Topic illustrate how immersive VR enables researchers to investigate sensorimotor function under standardized yet realistic conditions, providing new insights into balance regulation, motor adaptation, and their implications for athletic performance. For coaches and practitioners, these controlled environments also offer opportunities to assess movement quality and expose athletes to challenging training scenarios that may be difficult or unsafe to reproduce consistently in conventional practice.

Schedler et al. investigated the effects of age and arm movement condition on dynamic balance performance during virtual height exposure in children, adolescents, and young adults. Although unrestricted arm movements did not significantly influence balance performance, age-related differences emerged, with children performing worse than adolescents. Across all groups, balance performance improved markedly between trials, indicating rapid habituation to the immersive environment. These findings suggest that adaptation to VR occurs quickly and may mask expected motor control effects, highlighting the importance of accounting for habituation when interpreting balance performance in virtual settings. Summarizing experimental research in this field, Cariati et al. systematically reviewed randomized controlled trials evaluating VR-based balance training in athletes. Overall, VR interventions improved balance, postural stability, neuromuscular control, and sport-specific performance, although methodological heterogeneity limited direct comparisons. Focusing on injury prevention, Rahimi et al. systematically reviewed VR interventions targeting biomechanical and neuromuscular injury risk factors in athletes. Most studies reported improvements in movement mechanics, coordination, balance, and perceptual-cognitive control, but it remains unclear whether these short-term gains reduce injury risk.

Taken together, these contributions suggest that VR is an effective tool for assessing and training balance and sensorimotor function under highly controlled conditions. The available evidence indicates beneficial effects on various aspects of movement control; however, whether these functional improvements translate into enhanced athletic performance and reduced injury incidence remains a subject to future research. From a practical perspective, the current evidence suggests that VR is best viewed as a complementary training tool that can help coaches safely manipulate task difficulty, monitor sensorimotor adaptation, and supplement conventional balance and neuromuscular training rather than replace it.

## 3 Perceptual–cognitive training in immersive environments

Elite athletic performance depends not only on physical capabilities but also on the efficient integration of perception, attention, decision-making, and inhibitory control under time pressure. By combining immersive environments with precise experimental control, VR offers unique opportunities to investigate and train these perceptual-cognitive processes in sport-specific settings. At the same time, immersive technologies provide coaches with new possibilities to expose athletes to representative decision-making scenarios that are difficult to standardize or repeatedly reproduce in conventional training.

Another important theme emerging from this Research Topic is the application of immersive VR to the assessment and training of perceptual-cognitive skills that underpin athletic performance. Using a sample of healthy adults, Shimi et al. investigated attentional demands during a VR-based light-training task and demonstrated that performance depended on rapid attentional orienting and the ability to divide attention across multiple spatial locations, highlighting the cognitive demands underlying VR-based reaction training. Likewise, Bürger et al. examined perceptual-motor decision-making in healthy adults using a Go/No-Go paradigm in VR and found that the effects of stimulus eccentricity and inhibitory signals closely replicated established real-world findings, supporting the ecological validity of immersive VR for investigating attentional control and response inhibition. Extending these findings to an applied sporting context, Heilmann and Schubert evaluated a nine-week sport-specific VR intervention in elite youth ice hockey players. The intervention improved inhibitory performance in sport-specific flanker tasks, particularly under distracting conditions. However, effects did not transfer to general cognitive tests, suggesting that the benefits of VR-based cognitive training are largely domain-specific and depend on the similarity between training and competitive demands.

Taken together, these studies highlight the potential of VR as an ecologically valid platform for assessing and training perceptual-cognitive skills relevant to sport-specific performance. At the same time, they highlight an important challenge for future research: which cognitive adaptations extend beyond the virtual environment and translate into meaningful improvements in real-world performance? From a practical perspective, the current evidence suggests that coaches are likely to gain the greatest benefit from VR when training tasks closely replicate the perceptual and decision-making demands of competition, reinforcing the principle that specificity remains central to effective athlete development, regardless of the technology used.

## 4 VR, cognitive–motor interference, and performance modulation

While the previous studies demonstrate that VR can effectively assess and train perceptual-cognitive skills, successful athletic performance ultimately depends on integrating these cognitive processes with precise motor execution. The following contributions therefore examine how immersive VR influences cognitive–motor interactions, including dual-task performance and motor planning, under conditions that more closely resemble the complex demands of competitive sport. By recreating cognitively demanding movement scenarios under controlled conditions, VR also provides coaches and researchers with opportunities to investigate how athletes coordinate perception, cognition, and action during performance.

Two contributions examined cognitive–motor interactions in immersive VR from complementary perspectives. In elite youth ice hockey players, Brinkbäumer et al. compared acute VR-based and conventional dual-task training, showing that although both interventions improved simple motor speed, only conventional dual-task training enhanced cognitive–motor performance under linguistic processing demands. The greater interindividual variability observed following VR training suggests that its effectiveness depends on both task characteristics and athlete-specific factors. Complementing these findings, Schütz investigated motor planning during full-body movements in healthy adults performing tasks in real and virtual environments. Inverse hysteresis effects were evident under both conditions, indicating that previous motor plans influenced subsequent movement decisions. Additionally, participants adopted more cautious movement strategies in VR, suggesting that immersive environments also alter risk perception and motor behaviour.

Rather than focusing solely on training outcomes, these studies illustrate the broader value of VR as an experimental platform for studying cognitive–motor interactions. By enabling realistic yet highly standardized scenarios that are difficult to reproduce in the real world, immersive technologies can advance both mechanistic research and the development of targeted training interventions. For practitioners, these findings suggest that VR may be particularly valuable for designing progressive dual-task exercises and systematically manipulating cognitive demands while maintaining a safe and controlled training environment. However, the observed variability in training responses also highlights the need to individualize VR-based interventions according to athlete characteristics and task requirements.

## 5 Sport-specific simulation and mental skills training

One of the greatest strengths of VR is its ability to recreate sport-specific situations that are difficult, costly, or impractical to reproduce consistently in conventional training. Beyond physical skill acquisition, immersive simulations provide opportunities to develop perceptual, tactical, and psychological competencies within realistic yet highly controllable environments. For coaches, this creates opportunities to repeatedly expose athletes to representative performance scenarios while systematically manipulating task constraints that are often difficult to control in real-world practice.

Two contributions investigated how immersive VR can support sport-specific performance by combining realistic visual simulation with cognitive or psychological training. Witte et al. systematically reviewed technical and perceptual aspects of VR-based sports training across endurance, team, and individual sports. Their review highlights that effective interventions rely on targeted visual manipulations, such as altered perspectives, trajectory overlays, and cue highlighting, to enhance perceptual learning and decision-making. However, limitations in field of view, image resolution, and depth perception continue to restrict ecological validity and the transfer of training effects to real-world performance. Translating these principles into an applied setting, Cardenas Hernandez et al. evaluated immersive cognitive-behavioral training in long-distance runners. By practicing pacing strategies, drafting behavior, and motivational self-talk within virtual race environments, athletes improved strategy application, motivation, and, to a lesser extent, performance, illustrating that VR can support not only technical and tactical preparation but also the psychological skills underpinning endurance performance. Together, these findings demonstrate that VR can support not only technical and tactical preparation but also the psychological skills that underpin successful performance in endurance sports.

These two studies demonstrate that immersive VR simulation offers a more holistic approach to sport-specific training using by addressing technical, perceptual, tactical, and psychological components within a sport-specific training environment. As VR technology continues to evolve, improving simulation realism and visual fidelity will be critical for maximizing the transfer of these benefits to competitive sport.

## 6 Inclusion, accessibility, and special populations

Beyond performance enhancement, VR also has the potential to promote accessibility and inclusiveness in sport. Kirilova et al. evaluated the feasibility of immersive VR in six male athletes with mild to moderate intellectual disabilities participating in adapted basketball training. Across three VR sessions using basketball-specific 360° video content, athletes demonstrated positive emotional responses, minimal simulator sickness, stable balance performance, and high engagement, supporting the feasibility of VR as a safe and motivating supplementary training tool for persons with cognitive disability. These findings suggest that immersive VR may help reduce barriers to participation and support more individualized training. From a practical perspective, these findings highlight the potential of VR to support more inclusive coaching by providing adaptable training environments that can be tailored to the needs and abilities of individual athletes. As immersive technologies become more accessible, they may help broaden participation in sport while enabling coaches to deliver safe, engaging, and individualized training experiences across diverse athlete populations.

## 7 Conclusion

Collectively, the studies in this Research Topic demonstrate that VR has evolved from an experimental technology into an increasingly evidence-based tool for sports science. Across diverse applications, immersive technologies show considerable potential to enhance the assessment and training of perceptual-cognitive and motor skills while supporting more individualized and inclusive approaches to athlete development. However, technological limitations, heterogeneous methodologies, inconsistent transfer to real-world performance, and limited longitudinal evidence indicate that VR should be viewed as a complement rather than a replacement for conventional training.

Future research should focus more on causal, longitudinal studies, application of standardized protocols, individualized interventions, and direct comparisons between virtual and laboratory or real-world practice to determine when, how, and for whom VR provides meaningful performance benefits. At the same time, the current evidence suggests that immersive technologies can provide researchers and coaches with valuable opportunities to create representative, controlled, and adaptable training environments that go beyond the limits of conventional practice. Ultimately, the future impact of VR in sports will depend less on technological innovation than on robust scientific evidence guiding its effective application.

